# Experiences of informal caregivers supporting individuals diagnosed with bipolar disorder: a systematic review and thematic synthesis

**DOI:** 10.1186/s40345-025-00391-w

**Published:** 2025-10-27

**Authors:** Emily Roxburgh, Billie Lever Taylor, Aikaterini Rammou, Joanne Hodgekins

**Affiliations:** 1 Division of Methodologies, Florence Nightingale Faculty of Nursing, Midwifery and Palliative Care, King’s College London, London, UK; 2 Department of Clinical Psychology and Psychological Therapies, Norwich Medical School, University of East Anglia, Norwich, NR4 7TJ UK

**Keywords:** Bipolar disorder, Systematic review, Meta-synthesis, Thematic synthesis, Caregivers, Qualitative research

## Abstract

**Background:**

To identify, appraise, and synthesise qualitative studies exploring the experiences of informal caregivers (unpaid individuals providing emotional and or practical care) supporting individuals diagnosed with bipolar disorder (BD), and to identify any emotional, practical, or informational needs.

**Methods:**

Ovid, MEDLINE, Scopus, PsychINFO and CINAHL were searched from 1980 to January 2025*.* Studies were eligible for inclusion if they were peer viewed, published in English, used qualitative data collection and analysis, had data on the experiences of caregivers (aged 18 or above) supporting individuals with BD (aged 14 or above), and were conducted in western countries with individualistic cultures. Studies were appraised using the Critical Appraisal Skills Programme checklist. Data were analysed using thematic synthesis.

**Findings:**

Fourteen papers were included in the review. Three analytical themes: ‘challenges of caregiving’, ‘healthcare system challenges’, and ‘coping with the shifting landscape’ were identified, encompassing six descriptive themes and three supporting subthemes.

**Conclusions:**

Caregivers supporting individuals with BD face complex emotional and physical challenges, coupled with significant imposed losses and responsibilities. The relapsing and unpredictable nature of BD can exacerbate caregiver demands. There is a need for increased societal awareness of BD, improved communication and collaboration between mental health services and caregivers, and improved support for caregiver wellbeing. Further research exploring cultural, gender, and role specific needs of caregivers is warranted.

## Background

Bipolar disorders (BD), characterised by extreme changes in mood, affects over 1% of the world’s population (Grande et al. 2016; McIntyre et al. 2020). Whilst ‘stable’ periods can last weeks, months, or even years (Bipolar UK, 2021), subsyndromal symptoms between episodes are common (Grunze and Born 2020). BD follows a lifelong relapsing and remitting course, with unpredictable episodes making it difficult to manage for everyone involved (Hajda et al. 2016; Mignogna and Goes 2024). The aetiology of BD is complex, likely involving genetic, biological, psychological, interpersonal, societal and environmental factors (Goes, 2023; McIntyre et al. 2020), with onset often in adolescence or early adulthood (McGrath et al. 2023; Nowrouzi et al. 2016; Vieta et al. 2018). Early recognition and intervention are key for improving prognosis (Vieta et al. 2018). However, there is an average delay of approximately nine and a half years from the onset of symptoms to diagnosis (Bipolar UK, 2021; Fritz et al. 2017).

BD significantly impacts psychosocial functioning (Bonnín et al. 2019; MacQueen et al. 2001), with multiple episodes suggestive of poorer outcomes (Rosa et al. 2012). Compared to the general population, individuals with BD are at increased risk of physical health conditions, including diabetes, obesity, respiratory issues, and cardiovascular and kidney disease (Hayes et al. 2015; Kang et al. 2024; Nierenberg et al. 2023). They are also more likely to have comorbid psychiatric diagnoses, such as anxiety disorders, substance use, personality disorders, eating disorders, obsessive–compulsive disorder, and attention-deficit hyperactivity disorder (Altinbaş 2021; Carvalho et al. 2020; Krishnan et al. 2005). Additionally, the suicide rate among individuals with BD is approximately 20 times higher than that of the general population (Plans et al. 2019). A recent commission report highlights the need for improved monitoring, integrated care models, and tailored interventions to address these disparities and improve overall health outcomes (Firth et al. 2019).

Most guidelines recommend pharmacological treatment, including mood stabilisers (such as lithium) and or antipsychotic medication, for acute and long-term symptom management to prevent relapse and to stabilise mood (Connolly and Thase 2011; Goes 2023; NICE 2023). These are often combined with psychosocial treatments, such as psychological therapies like Cognitive Behavioural Therapy and family-focused interventions, and or lifestyle approaches (Goes 2023; NICE 2023; Vieta 2018). Beyond these treatments, informal caregivers (referred to as caregivers throughout this paper), usually unpaid family members, partners, or close friends, can provide emotional and or practical support to individuals with BD (Hannan 2013; House of Commons Library 2024; Lynch et al. 2018; NHS England 2025). Caregivers often assist with daily tasks, symptom monitoring, and treatment adherence, which can further reduce the risk of relapse and improve the quality of life for the individual with BD (Pompili et al. 2014; Van Den Heuvel et al. 2018). However, caregiving relationships can be complex and may also contribute to challenges for those with BD (Sharma et al. 2021; Ogilvie et al. 2005).

The significant role that caregivers play in the wellbeing of individuals with BD, often results in experiences of high levels of emotional, psychological, practical and financial stress (Bauer et al. 2011; Karambelas et al. 2022; Van Der Voort et al. 2007). This can lead to mental health difficulties such as depression and anxiety (Steele et al. 2010). Higher caregiver strain has been associated with the severity of the individual’s symptoms and the hours spent providing care (Mirhosseini et al. 2024; Saleh et al. 2013). Some caregivers feel they have no choice in taking on this role, with research suggesting that this alone could increase levels of emotional distress and poorer physical health outcomes (Schulz et al. 2012). Although caregiving can have positive aspects (Maskill et al. 2010; Veltman et al. 2002), it also involves many sacrifices and challenges, including financial, social, relational, and health impacts (Bauer et al. 2011; Guan et al. 2023; Hailegabriel and Berhanu 2023; Maskill et al. 2010; Seddigh et al. 2018; Speirs et al. 2023; Tranvåg and Kristoffersen 2008).

It is crucial to ensure that caregivers have access to appropriate resources, support, and are actively involved by services. Guidelines recommend that caregivers should receive psycho-education about BD, be involved in treatment and crisis planning, be provided with or signposted to psychological therapies and peer support, and if possible, respite care (APA 2002; Malhi et al. 2015; National Collaborating Centre for Mental Health 2006; NICE 2023; Yatham et al. 2018). This is particularly important as community-based and psychosocial care becomes more embedded in the UK and other parts of the world (NHS England 2019a; NHS England 2019b; WHO 2022). Therefore, there is a need to acknowledge the potential additional strains that caregivers may experience and better understand their needs.

Qualitative research offers a rich understanding of caregivers’ experiences of involvement and support from healthcare services. Many expressed feeling excluded from care processes, which sometimes led to distrust in healthcare professionals, and not being provided with psycho-education on BD or support for their own wellbeing (Baruch et al. 2018a; Bauer et al. 2011; Chatzidamianos et al. 2015; Richard-Lepouriel et al. 2022; Rusner et al. 2012; Shamsaei et al. 2010; Speirs et al. 2023). Despite the general dissatisfaction, some reported positive experiences where they were provided with information about BD and were involved in the individual’s care (Clements et al. 2019; Granek et al. 2018; Maskill et al. 2010; Tranvåg et al. 2008).

Although existing research has provided valuable insights into caregivers’ experiences, synthesising evidence allows for a more systematic and comprehensive understanding of their needs. Previous systematic reviews have focused on psychological interventions for caregivers (Baruch et al. 2018b), caregiver burden, coping, and support needs (Van Der Voort et al. 2007), or on understanding stigma (Latifian et al. 2023). However, none have explored the broader experiences of caregivers whilst capturing any nuances offered by employing a thematic synthesis approach. Therefore, this systematic review and thematic synthesis aims to systematically identify, appraise, and synthesise qualitative studies that explore the experiences among caregivers supporting individuals with BD, and to identify any emotional, practical, and or informational support needs.

## Methods

The current review was conducted in accordance with the Preferred Reporting Items for Systematic Reviews and Meta-Analyses (PRISMA) checklist (Page et al. 2021) and the Enhancing Transparency in Reporting the Synthesis of Qualitative research (ENTREQ) statement (Tong et al. 2012). The review protocol was developed collaboratively with the research team and registered on the Prospective Register of Systematic Reviews (PROPSERO) (reference number CRD42023487273).

### Search strategy

The SPIDER tool (Cooke et al. 2012) informed the free-text terms used in the search strategy, and the research questions. Compared to the PICO framework, the SPIDER tool is better suited for identifying qualitative papers due to its inclusion of ‘design’ and ‘research type’ (Methley et al. 2014). Incorporating these terms within ‘evaluation’ improved the retrieval of relevant qualitative papers. Boolean operators ‘AND’ and ‘OR’ were used to adjust the search scope, along with truncations for word variations. A librarian at the University of East Anglia reviewed the final search strategy for robustness, as found in Table 1. Searches were conducted by the first author on the 11th of December 2023 (and rerun on the 26th of January 2025 to account for any recent publications) across four electronic databases: Ovid MEDLINE, Scopus, PsychINFO and CINAHL. Searches were from 1980 to present (2025), reflecting the updated classification of BD in the DSM-III (Mason et al. 2016). Searches were limited to title and abstract, and peer reviewed journal articles in English. Phenomenon of interest was limited to title only.

**Table 1:** Final search strategy informed by the SPIDER tool (Cooke et al. 2012)

S-sample	Famil* OR caregiv* OR relative* OR partner* OR spouse AND
PI-phenomenon of interest	“Bipolar disorder” OR “mood disorder” OR “Bipolar” AND
D-design	(terms included in evaluation)
E-evaluation	Experience* OR perception* OR qualitative OR interview OR “mixed methods” OR “grounded theory” OR “thematic analysis” OR “interpretative phenomenological analysis” OR IPA OR “discourse analysis” OR “realist” OR “content analysis” OR “focus group”
R-research type	(terms included in evaluation)

### Inclusion and exclusion criteria

Studies were eligible if they had a qualitative or mixed-methods design and reported on the experiences of caregivers supporting an individual diagnosed with BD. For mixed-methods studies, only qualitative data were extracted. Studies including perspectives from other stakeholders or caregivers of individuals with different diagnoses (e.g., schizophrenia) were eligible, but only data clearly pertaining to caregivers of those with BD were extracted. If BD caregiver specific data were unclear, it was omitted.

Caregivers needed to be 18 years or older and either be a parent (where the individual with BD is aged 14 years or older, in line with the Early Intervention in Psychosis Services in England, NHS England 2023), adult child, sibling, spouse (or partner), other relative, close friend or neighbour. They also had to have a close relationship with the individual diagnosed with BD and or provide support (e.g., emotional, practical). If it was unclear who the caregivers were or whether they met the inclusion criteria, studies were either excluded or efforts were made to ensure that only relevant data were extracted.

Only studies conducted in western countries with individualistic cultures were included (Hofstede 1984). Given notable cultural differences in caregiving perceptions, healthcare systems, and mental health approaches, this decision was taken to focus on studies from similar cultural contexts and with similar mental health service provision to increase the applicability of review findings to clinical practice. A systematic approach was undertaken, with the first author and JH discussing each paper retrieved for full text screening (n = 50) until reaching consensus on its eligibility. It is recognised that some caregivers from collectivist cultures and or non-western origins may reside in western countries; any relevant cultural nuances were noted and synthesised where applicable.

Amendments to the protocol were made in March 2024, further information is available on PROSPERO (reference number CRD42023487273). These did not affect the search. See Table 2 for inclusion and exclusion criteria.

**Table 2:** Inclusion and exclusion criteria

Inclusion	Exclusion
Qualitative research studies exploring the views of informal caregivers over the age of 18 who identify as either a parent (to an individual aged 14 years or older), adult child, sibling, spouse (or partner), other relative, close friend or neighbour who have a close relationship and or provide substantial support to an individual diagnosed with bipolar disorder	Informal caregivers under the age of 18 years old
Individuals from western countries with individualistic cultures	Studies exploring the evaluation of interventions (e.g., support groups, programmes)
Qualitative component of mixed-method studies	Studies using only a quantitative research design
English language	Studies not available in the English language
Studies exploring the experiences of informal caregivers which encompass other stakeholders’ views	Studies reported in book chapters, conference papers, editorials, or letters
Studies exploring the experiences of informal caregivers which encompass bipolar disorder among other mental health conditions	Studies that do not include the views of informal caregivers of those who support an individual diagnosed with bipolar disorder
Studies published in peer-reviewed journals	Systematic reviews or thematic or meta-synthesis

### Screening

**Fig. 1:**
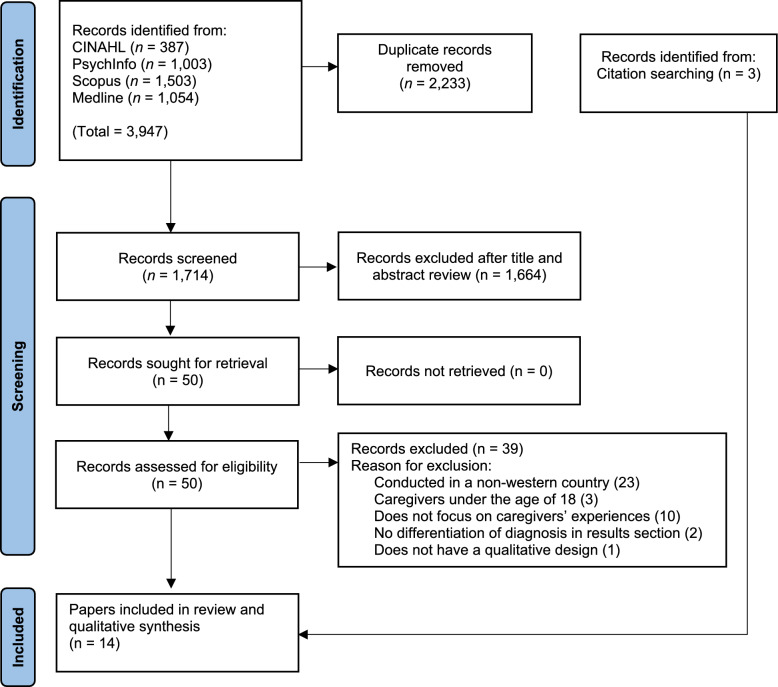
PRISMA flow diagram of study selection process

All records returned by the electronic database searches were imported into Zotero (version 6.0.37) and subsequently exported into Rayyan (Ouzzani et al. 2016) for deduplication and screening. The electronic searches identified 1714 records after deduplication, 1664 records were excluded following title and abstract screening and 39 records were excluded after full text screening, resulting in a total of 14 records, inclusive of three records (Rusner et al. 2013; Tranvåg and Kristoffersen 2008; Veltman et al. 2002) identified from backward citation searching of included papers. Forward citation searching was also performed in Google Scholar, revealing no relevant studies. See Fig. 1 for the PRISMA flow diagram. A second reviewer (AR) conducted full-text screening on 30% of the included papers, with any discrepancies discussed until a consensus was reached.

### Quality appraisal

The Critical Appraisal Skills Programme (CASP) checklist (2024) was used to assess the methodological quality of each paper. Recognised as a widely used tool in qualitative research (Long et al. 2020), the CASP checklist is also endorsed by the Cochrane Qualitative and Implementation Methods Group (Noyes et al. 2018). The first author rated each CASP criterion as “Yes”, “No”, or “Can’t tell”, with a second reviewer (AR) independently rating 30% of the papers; any discrepancies were discussed until consensus was reached. The “Can’t tell” rating was assigned to criteria lacking clear or sufficient information. The purpose of quality appraisal in this review was to assess the quality of available evidence on this topic. Studies were not excluded based on quality rating (Garside 2014), but quality and reliability of findings were considered in the synthesis.

### Data extraction

Data were extracted by the first author using a data extraction form created in Microsoft Excel, which was cross-checked by a second reviewer (JH), and piloted with two studies to ensure it effectively captured all relevant information (Long and Abraham 2016). This included contextual information of the studies, such as the author(s), year and country, aim(s), sampling method, population characteristics, method of data collection and analysis, and an overview of key findings (Table 3). All text under the results or findings section of the studies were extracted and organised into separate files, which were then imported into NVivo 14 Software to aid the synthesis.

**Table 3:** Characteristics of included studies

Author(s), year, and country	Aim(s)	Sampling method	Population characteristics	Data collection and analysis	Key themes
Baruch et al., (2018a), United Kingdom	To explore what challenges family members face in supporting a relative with bipolar disorder, and how they attempt to manage them	Sample recruited from voluntary sector organisations, university website, and through word of mouth	*N* = 18 Age: Mean = 48 (range 31 to 67) Gender: 14 females Ethnicity: 8 White British, 4 Other White, 4 Asian/Asian British, 2 Mixed Employment: not reported Relation: 6 parents, 5 spouses/partners, 3 siblings, 1 spouse and parent Time caring for: not reported Caregiver definition: close family member perceived themselves as providing substantial support	Semi-structured interviews Phenomenological Analysis	(1) Not knowing: “like being in a minefield” (2) It’s out of my control: “Sitting waiting for the next thing to happen” (3) “Treading on eggshells” (4) “Picking up on signs” (5) Times of crisis: “between a rock and a hard place” (6)“I have to make my voice heard”
Bauer et al., (2011), Germany	To qualitatively assess the burden on caregivers of patients with bipolar disorder	Sample recruited via person with bipolar disorder (inpatient) at a tertiary referral centre	*N* = 32 Age: Mean = 46.8 Gender: 22 females Ethnicity: not reported Employment: 26 full time/part time/apprenticeship, and 6 unemployed/retired Relation: 18 spouses, 7 parents, 3 children, 3 siblings, 1 friend Time caring for: not reported Caregiver definition: caregiver with whom they had the closest contact to	Semi structured interviews Content Analysis	(1) Lack of understanding patient’s behaviour, helplessness and hopelessness of the caregiver (2) Caregiver’s consternation resulting from the extent of patient’s suffering (3) Patient’s uncooperative behaviour and refusal of caregiver (4) Worries about the future (5) Burdened by specific symptoms of bipolar disorder (6) Switches between depression and mania burdensome
Chatzidamianos et al., (2015), United Kingdom	To explore the barriers and facilitators to relatives’ involvement in bipolar disorder	Self-referred volunteers. No further details available	*N* = *12* Age: Mean = 60 Gender: 7 females Ethnicity: 11 White British, 1 White Other Employment: 2 full time, 4 part time Relation: not reported Time caring for: not reported Caregiver definition: Had to be in regular contact and have emotional/practical supporting role in person’s life	Semi-structured interviews Framework Analysis	(1) Pre-existing world views (2) The quality of relationships and communication (3) Other structural impediments
Clements et al., (2019), United Kingdom	To explore relatives’ and service users’ experience of mental health services in relation to suicidal behaviour in bipolar disorder	Sample recruited via people who had presented at hospital with self-harm, via clinical studies officers in NHS, advertising via mental health charities and local newspapers, in-person visits to charities, and personal referrals from other participants/research colleagues	-	Semi-structured interviews Thematic Analysis	(1) Access to care (2) Problems with communication
Jönsson et al., (2011), Sweden	To elucidate what it means for family members to live with an adult person with bipolar disorder, with reference to their views concerning the condition of the person affected and the future	Sample recruited via person with bipolar disorder within outpatient services	*N* = 17 Age: Mean = 47 (range 28 to 82) Gender: 12 females Ethnicity: not reported Employment: 15 full time, 1 part time, 1 unemployed, 1 retired Relation: 10 parents, 5 married/cohabitating partner, 2 adult child Time caring for: not reported Caregiver definition: be regarded by person with bipolar disorder as significant influential person	Semi-structured interviews Content Analysis	(1) The family member’s view of the condition of the person with the illness (2) The family member’s view of their future
Maskill et al., (2010), New Zealand	To identify and gain a greater understanding of the way the lives of individuals have been both positively and negatively affected by supporting someone diagnosed with bipolar disorder	Sample recruited via advertisements being placed in local community, including government and non-government-based consumer services	*N* = 12 Age: range 30 to 65 + Gender: 11 females Ethnicity: not reported Employment: not reported Relation: 10 parents, 1 spouse, 1 sibling Time caring for: not reported Caregiver definition: spouse or equivalent, most frequent contact and involvement in treatment, be supporting financially, be emergency contact	Semi-structured interviews Thematic Analysis	(1) I am a much more compassionate person (2) It’s tough and It’s a sacrifice
Richard-Lepouriel et al., (2022), Switzerland	To explore the stigma-related experiences of family members, to explore how relatives perceive, experience and cope with stigma by association, and to assess whether coping differs in accordance with their roles in the family	Sample recruited from a mood disorder unit, either directly (via psycho-educational group for relatives) or indirectly (via person with bipolar disorder)	*N* = 12 Age: Mean = 53 (range 28 to 62) Gender: 8 females Ethnicity: not reported Employment: 28% professionals, 19% managers, 14% technicians, 24% retired, 10% unemployed, and one (5%) on disability insurance Time caring for: not reported Caregiver definition: not reported	Semi-structured interviews Thematic Analysis	(1) Language (2) Identity (3) Emotions (4) Others’ reactions (5) Coping with BD (6) Relative’s Needs
Rusner et al., (2012), Sweden	To elucidate the existential meaning of being closely related to a person with bipolar disorder	Not clearly defined	*N* = 12 Age: range 21 to 71 Gender: 6 females Ethnicity: not reported Employment: not reported Relation: 5 married/cohabitating, 4 parents, 2 siblings, and 1 adult child Time caring for: not reported Caregiver definition: adults closely related	Semi-structured interviews Phenomenological Analysis	(1) Struggling for survival (2) Having to compensate (3) Being both one step ahead and one step behind
Rusner et al., (2013), Sweden	To explore and describe what makes life as a close relative of a person with bipolar disorder more liveable	Not clearly defined	*N* = 12 Age: range 21 to 71 Gender: 6 females Ethnicity: not reported Employment: not reported Relation: 5 married/cohabitating, 4 parents, 2 siblings, and 1 adult child Time caring for: not reported Caregiver definition: adults closely related	Semi-structured interviews Phenomenological Analysis	(1) Essential meaning—influencing the unpredictable (2) Keeping a distance (3) Having stability (4) Strengthening equality through transparent communication
Speirs et al., (2023), Australia	To gain insight into the experiences of carers, and to better understand what help and information carers need	Sample recruited via social media advertising	*N* = 15 Age: Mean = 41 (range 20 to 60) Gender: 13 females Ethnicity: not reported Employment: not reported Relation: 7 spouses/partner, 5 parents, 2 adult children, 1 friend/housemate Time caring for: not reported Caregiver definition: main support person for individual with bipolar disorder	Semi-structured interviews Thematic Analysis	(1) Separation of the person and the disorder (2) Carer health and coping strategies (3) Unpredictability and variability of symptoms (4) Carer disillusionment and silencing (5) Story sharing and support needs
Tranvåg & Kristoffersen (2008), Norway	To explore the experiences of spouses/cohabitants who over time have lived with a partner who has bipolar disorder	Sample recruited from two psychiatric hospitals	*N* = 8 Age: Mean = 62 (range 31 to 85) Gender: 4 females Ethnicity: not reported Employment: not reported Relation: 8 spouses/cohabitants Time caring for: range from 6 to 51 years after onset of illness Caregiver definition: lives with partner and has experienced two episodes which led to psychiatric institution	Semi-structured interviews Phenomenological Analysis	(1) Experience formed part of a cumulative process (14 experiences) (2) Each experience creates a preunderstanding that affected how subsequent experiences were perceived (3) Preunderstanding also affected how they managed to master new illness-related challenges
Veltman et al., (2002), Canada	To gain a greater knowledge of the meaning that caregivers derive from their situation, including positive aspects	Sample recruited via advertisements in teaching hospital, through local mental health organisations, and family support groups	-	Semi-structured interviews Analysis not reported	(1) Stigma (2) Systems issues (3) Life lessons learned (4) Love and caring for the ill relative
Van Den Heuvel et al., (2018), The Netherlands	To describe how caregivers have learned to overcome the impact of emotional burden and experienced distress, and how they have developed a personal definition of efficient self-management support for individuals living with bipolar disorder	Sample recruited by asking individuals with bipolar disorder who were part of a parallel study to identify caregiver	*N* = 10 Age: Mean = 52 Gender: 7 females Ethnicity: not reported Employment: not reported Relation: 5 spouses, 2 parents, 1 sibling, 1 friend, 1 adult child Time caring for: Mean = 11.5 years Caregiver definition: significant person who support them in self-management activities	Semi-structured interviews Phenomenological Analysis	(1) Understanding bipolar disorder (2) Overcoming the dilemmas in self-management support for individuals living with bipolar disorder (3) Dividing tasks and responsibilities (4) Acquiring a personal definition of self-management support for individuals living with bipolar disorder
Van Der Voort et al., (2009), The Netherlands	To gain a greater insight into experienced burden, coping mechanisms, and the support needs of spouses	Sample recruited during an annual meeting for spouses of those with bipolar disorder, via an online advertisement posted on a website for single parents, and via newspaper advertisements	*N* = 15 Age: Mean = not reported (range 30 to 60) Gender: 9 females Ethnicity: not reported Employment: not reported Relation: 11 spouses, 4 ex-spouses Time caring for: not reported Caregiver definition: Spouse of at least 5 years who lives with the person with bipolar disorder	Semi-structured interviews Grounded Theory	(1) Burden (2) Appraisal (3) Self-effacement versus self-fulfilment (4) Mediating factors (5) Resignation

### Data synthesis

Findings were analysed using Thomas and Harden’s (2008) thematic synthesis approach, which involves three steps: First, line-by-line coding, where codes were applied to each line of data across all papers. Data were applied to existing codes, or new codes were created as needed. Second, descriptive themes were developed by grouping related codes across papers, they remained close to the original meaning of the primary data. Third, analytical themes were generated to interpret deeper meanings, taking the synthesis beyond the primary data to address the research questions proposed in this review using an inductive approach (Thomas and Harden 2008). Data synthesis was undertaken by the first author, with ongoing discussions with reviewers (JH & BLT) to ensure a triangulation approach throughout the synthesis, which led to framework adaptations.

## Findings

### Study characteristics

The 14 studies included in the review represented the views of over 163 caregivers, most of whom were female (N @ 119). Clements et al. (2019) and Veltman et al. (2002) included additional stakeholders and caregivers of individuals with other diagnoses, making it difficult to isolate the number of caregivers specific to individuals with BD. Rusner et al.’s (2012, 2013) studies shared the same dataset but explored different aims.

Participants were recruited through various methods, and the studies were conducted across the United Kingdom, (n = 3), Australia, Germany, Switzerland, The Netherlands (n = 2), Sweden (n = 3), New Zealand, Norway and Canada. Participants’ ages ranged from 20 to 85 years, with several studies reporting average ages only (Bauer et al. 2011; Chatzidamianos et al. 2015; Clements et al. 2019; Van den Heuvel et al. 2018). Ethnicity was reported in only two studies, time spent caring for the individual in two, and employment status in four. Rusner et al. (2012, 2013) noted that one participant had immigrated to Sweden from an Asian country, whilst another was raised by immigrant parents from South America.

All studies, except Clements et al. (2019), reported the caregiver’s relationship to the individual with BD. Spouses/partners (n = 74) were the most frequently reported, followed by parents (n = 49), siblings (n = 14), adult children (n = 12) and friends (n = 3). Different methods of reporting caregiver relationship in some studies (Chatzidamianos et al. 2015; Clements et al. 2019; Rusner et al. 2013; Veltman et al. 2002) made it challenging to summarise the overall number of caregivers by relationship. Although all studies met the inclusion criteria, definitions of informal caregiver varied across studies, thus reflecting differences in how caregiving is conceptualised.

Data were collected through semi-structured interviews and analysed using thematic analysis (Clements et al. 2019; Maskill et al. 2010; Richard-Lepouriel et al. 2021; Speirs et al. 2023), content analysis (Bauer et al. 2011; Jönsson et al. 2011), phenomenological analysis (Baruch et al. 2018a; Rusner et al. 2012, 2013; Tranvåg and Kristoffersen 2008; Van Den Heuvel et al. 2018), framework analysis (Chatzidamianos et al. 2015), and grounded theory (Van Der Voort et al. 2009).

### Quality assessment

See Table 4 for CASP ratings. All 14 studies employed appropriate qualitative methodologies, clearly stated their aims, and aligned their research designs with these aims. Recruitment strategies were appropriate to the aims of the research in all studies except for Jönsson et al. (2011), where it was unclear how participants were invited to take part. Data collection methods in all studies effectively addressed the research aim. However, seven studies provided limited and or unclear information on considerations regarding the potential influence of the researcher’s background and how this might introduce bias into the research processes. Ethical issues were taken into consideration in 13 studies; however, Richard-Lepouriel et al. (2022) would have benefitted from specifying how the research was explained to participants to assess whether ethical standards were maintained. All studies provided clear statement of findings, although Veltman et al. (2002) did not outline the type of data analysis undertaken. Nonetheless, 13 studies demonstrated sufficiently rigorous data analysis, and all studies were considered valuable contributions to research.

**Table 4:** Quality assessment of included papers

First author	Aims	Qualitative Methodology Appropriateness	Design	Recruitment	Data Collection	Researcher relationships	Ethics	Analysis	Findings	Value
Baruch (2018a)	Yes	Yes	Yes	Yes	Yes	Yes	Yes	Yes	Yes	Yes
Bauer (2011)	Yes	Yes	Yes	Yes	Yes	Can’t tell	Can’t tell	Yes	Yes	Yes
Chatzidamianos (2015)	Yes	Yes	Yes	Yes	Yes	Yes	Yes	Yes	Yes	Yes
Clements (2019)	Yes	Yes	Yes	Yes	Yes	Yes	Yes	Yes	Yes	Yes
Jönsson (2011)	Yes	Yes	Yes	Can’t tell	Yes	Can’t tell	Yes	Yes	Yes	Yes
Maskill (2010)	Yes	Yes	Yes	Yes	Yes	Can’t tell	Yes	Yes	Yes	Yes
Richard-Lepouriel (2022)	Yes	Yes	Yes	Yes	Yes	Can’t tell	Yes	Yes	Yes	Yes
Rusner (2012)	Yes	Yes	Yes	Yes	Yes	Can’t tell	Yes	Yes	Yes	Yes
Rusner (2013)	Yes	Yes	Yes	Yes	Yes	Can’t tell	Yes	Yes	Yes	Yes
Speirs (2023)	Yes	Yes	Yes	Yes	Yes	Yes	Yes	Yes	Yes	Yes
Tranvåg (2008)	Yes	Yes	Yes	Yes	Yes	Yes	Yes	Yes	Yes	Yes
Veltman (2002)	Yes	Yes	Yes	Yes	Yes	Can’t tell	Yes	Yes	Yes	Yes
Van Den Heuvel (2018)	Yes	Yes	Yes	Yes	Yes	Yes	Yes	Yes	Yes	Yes
Van Der Voort (2009)	Yes	Yes	Yes	Yes	Yes	Yes	Yes	Yes	Yes	Yes

### Thematic synthesis

Three analytical themes were identified, encompassing a total of six descriptive themes, with three supporting subthemes (Table 5). The analytical themes were ‘challenges of caregiving’, ‘healthcare system challenges’, and ‘coping with the shifting landscape’.

**Table 5:** Analytical themes with supporting descriptive themes and subthemes of included papers

	Baruch (2018a)	Bauer (2011)	Chatzidamianos (2015)	Clements (2019)	Jönsson (2011)	Maskill (2010)	Richard-Lepouriel (2022)	Rusner (2012)	Rusner (2013)	Speirs (2023)	Tranvåg (2008)	Veltman (2002)	Van Den Heuvel (2018)	Van Der Voort (2009)
Analytical Theme 1: Challenges of Caregiving	√	√			√	√	√	√	√	√	√	√	√	√
The weight of care		√				√	√	√	√	√		√	√	√
Coming to terms	√	√				√	√	√	√	√	√	√		√
The hidden costs		√			√	√	√	√	√	√	√	√		√
Alone in the struggle		√			√		√	√			√	√		√
Analytical Theme 2: Healthcare System Challenges	√	√	√	√		√	√	√	√	√	√	√	√	√
Left out of the loop	√	√	√	√		√	√	√	√	√	√		√	
Barriers at every turn		√	√	√		√		√		√		√		√
What we need	√			√			√		√	√				√
Analytical Theme 3: Coping with the Shifting Landscape	√	√	√	√	√	√	√	√	√	√	√		√	√
Riding the rollercoaster	√	√	√	√	√	√	√	√	√	√	√		√	√
Living on high alert	√	√					√	√		√	√		√	

### Challenges of caregiving

The first analytical theme, comprising one descriptive theme, and three subthemes, captures the multifaceted impacts on caregivers when supporting individuals with BD. Caregiving demands reshape their daily lives and can affect their mental and physical wellbeing. This is often accompanied by isolation from friends and family, sometimes worsened by stigma and others’ judgements. Despite these challenges, caregivers strive to find coping strategies and sustain hope for the future.

### Descriptive theme: the weight of care

Being responsible meant ensuring the wellbeing of the person with BD, including monitoring their symptoms, managing medication, overseeing the household, and or childcare duties (Bauer et al. 2011; Maskill et al. 2010; Rusner et al. 2012; Van Der Voort et al. 2009).

If I happen to make a mistake…everything is going to collapse…this responsibility is very heavy (Van Der Voort et al. 2009, p. 437).

Caregivers commonly sacrificed time for self-care, leisure, and family connections (Bauer et al. 2011; Maskill et al. 2010; Rusner et al. 2012; Veltman et al. 2002). The chronic nature of BD enforced a ‘forever strain’ of responsibility, often causing hopelessness, frustration, and a need for respite to continue (Maskill et al. 2010; Rusner et al. 2012; Veltman et al. 2002). One parent noted that the diagnosis made them realise they would be responsible for their child ‘until the day they die’ (Maskill et al. 2010, p. 539).

So what we’re talking about is the duration…say a manic spell or whatever… you get over it…but when it comes back and it’s again, and it’s again…it just gets pretty oppressive. This is not year one, this is year twenty one and I am exhausted (Maskill et al. 2010, p. 539).

This sense of responsibility often disrupted caregivers’ work lives, with some having no option but to leave their employment or reduce their hours, leading to financial strain (Bauer et al. 2011; Rusner et al. 2012; Van Der Voort et al. 2009). This was further exacerbated when individuals engaged in manic overspending (Bauer et al. 2011). Caregiving responsibilities often shifted family dynamics, with children adopting parental roles (Rusner et al. 2012; Speirs et al. 2023), spouses taking on additional roles (Bauer et al. 2011; Van Der Voort et al. 2009), and siblings taking on mediating roles (Richard-Lepouriel et al. 2022; Van Den Heuvel et al. 2018). Some caregivers managed their responsibilities by encouraging self-management and negotiating tasks, whilst others felt that distance was necessary (Rusner et al. 2013; Van Den Heuvel et al. 2018).

I told him ‘I am so tired and sad and have no energy left due to taking care of you. I need peace and quiet and have to get some more energy for myself’…I told him that I am still his girlfriend but I need to move out from the apartment (Rusner et al. 2013, p. 165).

Many also highlighted positives, such as increased empathy, patience, and or deeper sense of love for the person with BD (Maskill et al. 2010; Rusner et al. 2012; Speirs et al. 2023), which made their role feel less burdensome.

### Subtheme: the hidden cost

Caregivers experienced a complex range of emotions, such as worry, stress, guilt, hopelessness, and grief over what BD had taken from both the individual and themselves (Bauer et al. 2011; Maskill et al. 2010; Speirs et al. 2023; Tranvåg et al. 2008; Veltman et al. 2002).

It’s just totally depressing, it’s awful. Hopelessness for him and hopelessness for me. Thinking of him living a life that’s so dysfunctional and so often unhappy and lonely (Maskill et al. 2010, p. 538).

They also struggled with feelings of anger, shame, and despair because of the ‘BD label’ and burdens they faced (Richard-Lepouriel et al. 2022; Tranvåg et al. 2008). Many caregivers experienced profound loneliness, even during ‘stable’ periods (Rusner et al. 2012; Tranvåg et al. 2008; Van Der Voort et al. 2009).

It is not possible to be in contact with him…no matter how hard you want to…that causes so much grief and pain… the being alone (Van Der Voort et al. 2009, p. 437).

This emotional strain often resulted in new or worsening mental health difficulties, such as anxiety and depression (Speirs et al. 2023). Some caregivers also experienced physical health difficulties, including weight gain, muscular pain, tension, and fatigue (Bauer et al. 2011; Speirs et al. 2023; Tranvåg et al. 2008; Van Der Voort et al. 2009). Uncertainty about their capacity to provide adequate care added further stress (Tranvåg et al. 2008). Some spouse (or partner) caregivers ended or considered ending their relationship, although this was not an option for parents (Richard-Lepouriel et al. 2022; Rusner et al. 2012; Van Der Voort et al. 2009).

‘I’m fed up of living like this, but I love him, I’m staying anyway,’ the fact of staying leads us to start asking ourselves ‘But do I respect myself?.’ Somebody else would leave in two seconds (Richard- Lepouriel et al. 2022, p. 181).

A father can’t cut his ties with his son, it’s unthinkable. You can’t let your children down (Rusner et al. 2012, p. 203).

For many, the inability to express their needs exacerbated loneliness and stress, although some found helpful ways to navigate this (Baruch et al. 2018a; Rusner et al. 2012, 2013; Van Der Voort et al. 2009).

Many times I couldn’t stop crying. I didn’t protect him in showing my feelings…And then because I was really low we somehow will reverse position…he was looking after me…which made him feel good. (Baruch et al. 2018a, p. 1127).

Some caregivers found relief through psychological or pharmacological support, or substances, whilst others managed by setting boundaries, creating personal space, or implementing self-care practices (e.g., exercise, meditation, hobbies) (Richard-Lepouriel et al. 2022; Rusner et al. 2013; Speirs et al. 2023; Tranvåg et al. 2008; Van Der Voort et al. 2009). Reflecting alone or with others also helped reduce their emotional burden and provided moments of relief (Rusner et al. 2013; Van Der Voort et al. 2009).

### Subtheme: alone in the struggle

Caregivers often experienced significant social isolation, with many withdrawing or cancelling plans due to the demands of their role and the unpredictable nature of BD (Bauer et al. 2011; Maskill et al. 2010; Richard-Lepouriel et al. 2022; Rusner et al. 2012; Tranvåg et al. 2008). Many felt misunderstood and unsupported by friends and family, particularly during crisis or hospital admissions (Richard-Lepouriel et al. 2022; Rusner et al. 2012; Speirs et al. 2023; Tranvåg et al. 2008).

Our existence is very lonely. We can’t visit anyone, and it’s very seldom that anyone drops in on us. It isn’t so easy to leave her to visit mates…sometimes I drive down to the harbour and look for familiar faces when she has gone to sleep at night (Tranvåg et al. 2008, p. 9).

This lack of support was worsened by friends’ and families’ limited understanding of BD, with caregivers frequently at the receiving end of unhelpful responses (Jönsson et al. 2011; Maskill et al. 2010; Speirs et al. 2023; Tranvåg et al. 2008). The stigma surrounding BD heightened feelings of social isolation, especially when the individual displayed visible symptoms (Jönsson et al. 2011; Richard-Lepouriel et al. 2022; Van Der Voort et al. 2009). Some caregivers reframed reasons for hospitalisations to avoid stigma (Richard-Lepouriel et al. 2022). They also felt their caregiving role was misunderstood, with others failing to appreciate its complexities (Richard-Lepouriel et al. 2022; Veltman et al. 2002).

I have friends who don’t respect what I do, they don’t understand it, they feel sorry for me, they’re so prepared to be sympathetic. There’s an assumption that is made that there’s no quality of life for a caregiver, that it can’t possibly be rewarding or interesting (Veltman et al. 2002, p. 110).

The lack of social connection increased caregivers’ feelings of strain, leading some to dedicate more attention to the individual with BD (Rusner et al. 2012). Hypervigilance to protect the individual from stigma further added to their responsibilities (Rusner et al. 2012). Caregivers called for greater societal awareness of BD to reduce stigma and discrimination, making the diagnosis easier to manage (Richard-Lepouriel et al. 2022; Veltman et al. 2002). Whilst most found it difficult, some were able to talk to friends and family about their difficulties, providing relief and strength to continue (Jönsson et al. 2011; Richard-Lepouriel et al. 2022). Some friends and family offered visits or practical support, which caregivers welcomed (Maskill et al. 2010; Rusner et al. 2013; Van Der Voort et al. 2009).

### Subtheme: coming to terms

Caregivers found that adjusting their perspective was essential for acceptance (Jönsson et al. 2011; Van Der Voort et al. 2009; Veltman et al. 2002). Many recognised a ‘new normal’, shifting from an idealised expectation to acceptance of how things were, which provided inner peace (Jönsson et al. 2011; Rusner et al. 2012; Tranvåg et al. 2008).

Different things happen with my father…you automatically get used to…how he answers the phone, what he’s like when he comes over…these things become part of your everyday life…I do not find anything complicated…you just live with it; it’s that simple because it’s my dad (Jönsson et al. 2011, p. 32).

Caregivers often made an active choice to accept the individual’s diagnosis (Tranvåg et al. 2008; Van Der Voort et al. 2009). Others maintained a positive attitude, appreciated small moments, and focused on being present (Tranvåg et al. 2008; Veltman et al. 2002). These approaches fostered acceptance and a more balanced view of life with an individual with BD.

Living successfully with a family member with a mental illness means living unconventionally. You can’t worry about tomorrow too much…I’ve learned to cherish every day…every relationship, and I look at life very differently now (Veltman et al. 2002, p. 112).

Hope was essential for caregivers (Tranvåg et al. 2008; Richard-Lepouriel et al. 2022). They believed in the potential for improvement and stability in the individual’s condition (Jönsson et al. 2011). Many recognised the need to step back and allow the individual autonomy, aiming for a balance in responsibilities (Richard-Lepouriel et al. 2022; Van Der Voort et al. 2009). However, some struggled with the urge to always be present (Jönsson et al. 2011), alongside concerns about who would care for them in the future (Bauer et al. 2011; Jönsson et al. 2011; Rusner et al. 2012; Veltman et al. 2002).

### Healthcare system challenges

The second analytical theme, consisting of three descriptive themes, highlights the barriers caregivers encountered within the healthcare system. Challenges included collaborating with healthcare professionals and navigating certain service structures. Caregivers noted gaps in services failing to meet their needs and emphasised the need for improvements.

### Descriptive theme: left out of the loop

Caregivers often feel excluded from care processes, especially during inpatient stays (Baruch et al. 2018a; Bauer et al. 2011; Chatzidamianos et al. 2015; Rusner et al. 2012, 2013). Caregivers were often privy to key information about the individual. They felt frustrated when healthcare professionals overlooked their input, especially when lacking understanding of the individual’s background (Chatzidamianos et al. 2015; Clements et al. 2019; Maskill et al. 2010; Rusner et al. 2013).

When he died…the psychiatrist had actually said that he didn't think it was planned suicide…even though he had been telling me…he didn't want to live (Clements et al. 2019, p. 7).

Caregivers frequently encountered dismissive attitudes from healthcare professionals, who sometimes made decisions regarding the individuals’ care without their input, leaving caregivers feeling like outsiders (Baruch et al. 2018a; Bauer et al. 2011; Rusner et al. 2012; Tranvåg et al. 2008). This exclusion left caregivers feeling like ‘the problem’, leading to further desperation. This was particularly problematic after inpatient discharges when caregivers felt they were left with little information on what to do (Tranvåg et al. 2008).

I have almost no communication with the people treating her. I feel as if they’re saying: ‘You’re an outsider, we’re the professionals, you must just stay out of it’. Nobody tells me how we are supposed to handle this after her discharge…I have a bag full of medicines I’m supposed to give her. That’s the support apparatus we have (Tranvåg et al. 2008, p. 9).

Limited information from healthcare professionals regarding the individual’s diagnosis, treatment, and prognosis resulted in caregivers seeking out information online (Baruch et al. 2018a; Bauer et al. 2011; Richard-Lepouriel et al. 2022; Speirs et al. 2023; Tranvåg et al. 2008; Van Den Heuvel et al. 2018). They also felt that healthcare professionals missed opportunities to check in on their wellbeing (Speirs et al. 2023).

I’m genuinely surprised that there was no mention or even a question of, how are you coping? Are you doing okay? Is there any support that we can give you?…if that person supporting them isn’t doing the best or doesn’t know how to handle the circumstance, it’s like a recipe for disaster (Speirs et al. 2023, p. 10).

Some caregivers highlighted services’ openness to their involvement, which sometimes improved over time (Baruch et al. 2018a; Maskill et al. 2010; Tranvåg et al. 2008). They valued opportunities to discuss concerns and the forthcoming nature of healthcare professionals (Baruch et al. 2018a; Richard-Lepouriel et al. 2022).

### Descriptive theme: barriers at every turn

Caregivers reported systemic, organisational, and institutional barriers when seeking support, adding to their frustration. Accessing appropriate mental health services was complex and confusing, especially during crises (Clements et al. 2019; Maskill et al. 2010; Rusner et al. 2012; Van Der Voort et al. 2009). Services for people with BD felt limited (Maskill et al. 2010). Admission to inpatient care was difficult and often followed by quick discharges, even when caregivers believed the individual was still unwell (Rusner et al. 2012). This was particularly challenging when caregivers felt there was a gap between acute crisis and ‘stable’ in terms of support available (Maskill et al. 2010). Caregivers also felt that obtaining updates and information was hindered by dysfunctional systems for sharing information (Rusner et al. 2012).

I rang about the medicine for my daughter, and it’s not just about ringing to the doctor and asking, you have to first ring to the secretary, and then the secretary rings to the nurse, and then you have to wait for the nurse to ring you, and then you can talk to the nurse who approves that you can speak to the doctor (Rusner et al. 2012, p. 202).

Patient confidentiality posed another barrier, especially for parents whose child had turned 18, as they felt excluded from information sharing and decision making (Clements et al. 2019; Rusner et al. 2012). Some caregivers also encountered barriers when managing the individual’s finances (Rusner et al. 2012; Veltman et al. 2002).

I can’t control how he spends his money…there is nothing in the system to let a wife take charge of the finances (Veltman et al. 2002, p. 111).

Many expressed dissatisfactions with the treatment and support provided to the individual with BD, citing issues with medication and misdiagnoses (Bauer et al. 2011; Maskill et al. 2010; Rusner et al. 2012; Speirs et al. 2023). Caregivers felt disheartened by the lack or refusal of guidance and signposting from services on accessing additional support for themselves, even when they were willing to pay (Clements et al. 2019). They felt let down by a system that seemed unresponsive to both their needs and those of the individual with BD, emphasising a need for organisational change at all levels (Chatzidamianos et al. 2015).

### Descriptive theme: what we need

Caregivers advocated for improved policies and collaborative practices between mental health services and caregivers (Richard-Lepouriel et al. 2022; Rusner et al. 2013; Speirs et al. 2023). Many expressed a desire to be more involved in care discussions, decision making, and crisis planning (Baruch et al. 2018a; Clements et al. 2019; Speirs et al. 2023). They also wanted to feel heard and have a space to express their emotions (Richard-Lepouriel et al. 2022).

The person who are close to a person with BD…they also need to be listened to, probably much more than the patient himself…they are the ones who are there with her everyday (Richard-Lepouriel et al. 2022, p. 188).

Healthcare professionals were seen as crucial in establishing transparent communication between caregivers and individuals with BD (Rusner et al. 2013). Caregivers recognised the key role of healthcare professionals in fostering joint responsibility, supporting caregiver involvement, and strengthening relationships (Rusner et al. 2013; Van Der Voort et al. 2009). They also emphasised the need for healthcare professionals to share information about BD symptoms, treatment, prognosis, and crisis management (Speirs et al. 2023; Van Der Voort et al. 2009).

The psychiatrist should invite the partner or children for a meeting and ask how they are doing, then they get information some patients do not give (Van Der Voort et al. 2009, p. 440).

They also expressed a desire for psychological skills and coping strategies to manage stress, practice self-care, set boundaries, and improve communication with the individual (Baruch et al. 2018a; Richard-Lepouriel et al. 2022; Speirs et al. 2023; Van Der Voort et al. 2009). Some caregivers called for places of respite to ‘recharge their batteries’ (Richard-Lepouriel et al. 2022).

### Coping with the shifting landscape

The third analytical theme, comprising two descriptive themes, outlines the challenges caregivers face with the shifting nature of BD. Unpredictable and variable symptoms were difficult to navigate, leaving caregivers to feel constantly alert, further intensifying their distress. Some developed coping techniques that helped them manage.

### Descriptive theme: riding the rollercoaster

Caregivers described the episodic nature of BD as being on a rollercoaster (Baruch et al. 2018a; Rusner et al. 2012; Speirs et al. 2023). Initial reactions to the diagnosis were filled with shock, fear, and confusion as they struggled to comprehend and manage the significant changes in the individual (Baruch et al. 2018a; Bauer et al. 2011; Jönsson et al. 2011; Richard-Lepouriel et al. 2022; Tranvåg et al. 2008).

His behaviour was abnormal, completely different from the modest person we knew before. He turned into being supercilious and overconfident, and later…he became very timid and hesitant (Van Den Heuvel et al. 2018, p. 533).

Some caregivers described a feeling of ‘treading on eggshells’, unsure what to say or do during BD episodes (Baruch et al. 2018a; Speirs et al. 2023; Tranvåg et al. 2008; Van Den Heuvel et al. 2018). Transparent communication with the individual helped several caregivers better navigate BD’s unpredictability (Rusner et al. 2013; Speirs et al. 2023). Others emphasised the importance of having knowledge about BD, understanding what to expect during and after episodes, and trusting the individual’s ability to cope as essential for managing uncertainty and regaining hope and control (Baruch et al. 2018a; Van Den Heuvel et al. 2018). During manic episodes, caregivers often felt excluded and struggled with the out of character behaviours such as overspending, aggression, hurtful accusations, risky behaviours and sexual indiscretions (Baruch et al 2018a; Bauer et al. 2011; Chatzidamianos et al. 2015; Jönsson et al. 2011; Maskill et al. 2010; Rusner et al. 2012; Speirs et al. 2023; Tranvåg et al. 2008).

His whole personality changed completely when the mania came. It was as though he was possessed by something alien. He ‘disappeared’ and ‘someone else’ took over…It was so frightening because I didn’t understand….my brain was put out of action, had nothing to offer (Tranvåg et al. 2008, p. 8).

Many noted the need to mentally separate the person from the diagnosis to maintain a relationship (Richard-Lepouriel et al. 2022; Speirs et al. 2023; Van Der Voort et al. 2009). During depressive episodes, caregivers often felt useless and overwhelmed by the weight of the individual’s despair (Bauer et al. 2011; Rusner et al. 2012; Van Der Voort et al. 2009). These episodes brought worry about safety, powerlessness, and stress, which intensified during hospitalisations (Baruch et al. 2018a; Rusner et al. 2012; Speirs et al. 2023; Van Den Heuvel et al. 2018). The variability of symptoms caused heightened stress, vigilance, and exhaustion, as caregivers never knew when the next episode might occur and frequently felt one step behind (Baruch et al. 2018a; Bauer et al. 2011; Rusner et al. 2012, 2013; Tranvåg et al. 2008).

I experience the bipolar “emotional storms”…life is also a roller coaster for me…he’s in the first carriage and experiences the up and down immediately. I am at the back and am perhaps still at the top when he rushes down and I don’t understand at all what has happened because “everything” was fine…then I am still tired and sulky because I can’t really get to him when he is on the way up again and thinks that I am boring and doesn’t understand how good life is. It is not often that the train goes horizontally in a rollercoaster so we misunderstand each other all too often (Rusner et al. 2012, p. 205).

Some felt the individual’s lack of insight into their diagnosis and or non-compliance with treatment compounded challenges (Bauer et al. 2011; Van Den Heuvel et al. 2018). One study highlighted how manic behaviours could be interpreted differently dependant on cultural contexts (Rusner et al. 2012). Several caregivers found that creating distance from BD specific situations allowed them to reflect and regain a sense of control (Richard-Lepouriel et al. 2022; Rusner et al. 2013). Maintaining routines, engaging in activities, and peer support also helped manage the unpredictable (Richard-Lepouriel et al. 2022; Rusner et al. 2013; Van Den Heuvel et al. 2018).

### Descriptive theme: living on high alert

Caregivers described living in a state of hypervigilance, constantly monitoring for sudden and or unexpected changes in the individual’s symptoms (Richard-Lepouriel et al. 2022; Rusner et al. 2012; Tranvåg et al. 2008; Van Den Heuvel et al. 2018).

I pay attention to everything because I’m careful that it does not go too high or too low, therefore I’m always a bit unstable…I’m always a bit in control (Richard-Lepouriel et al. 2022, p. 187).

This heightened vigilance was often a result of the unpredictable and variable nature of BD. Caregivers explained that this alertness sometimes created a sense of ‘stability’ amidst chaos, although it was fragile and often led to a near-constant fight or flight state (Rusner et al. 2012; Speirs et al. 2023).

There’s such a chaotic situation here…which puts an even greater pressure on me never to be ill, never be aggressive, never act out…always be the stable person. That pressure is like a large bubble that makes it ache in my chest and threatens to burst (Rusner et al. 2012, p. 204).

Many caregivers tried to prevent BD episodes whilst respecting the individual’s autonomy (Baruch et al. 2018a; Rusner et al. 2012; Van Den Heuvel et al. 2018). Some hesitated to suggest treatment, fearing that it would be perceived negatively (Baruch et al. 2018a; Rusner et al. 2012; Van Den Heuvel et al. 2018). Others developed communication strategies to facilitate these conversations (Baruch et al. 2018a; Van Den Heuvel et al. 2018). Caregivers became skilled at recognising early signs of episodes, sometimes before the individual was aware, although they sometimes struggled to differentiate between normal and abnormal behaviours (Van Den Heuvel et al. 2018).

When we read the information leaflet that summed up the features of BD, we recognised some things…symptoms appeared insidiously…that is the elusiveness of this disease; you never know when an episode will recur…that is why… if there is a sale at a store and he buys a thing or two…I have to be alert. Just in case (Van Den Heuvel et al. 2018, p. 535).

This chronic state of alertness left caregivers feeling perpetually on edge and burdened by the weight of responsibility.

## Discussion

To our knowledge, this is the first review of qualitative studies to synthesise the experiences of caregivers supporting individuals diagnosed with BD. From this, three analytical themes were identified: ‘challenges of caregiving’, ‘healthcare system challenges’, and ‘coping with the shifting landscape’.

Similar to a previous review (Van Der Voort et al. 2007), this review reveals the significant responsibility for providing support placed on caregivers, along with the associated challenges and losses. They experience emotional and physical health impacts, often accompanied by new or worsening mental health difficulties. These feelings often led caregivers to question their role and whether to stay or leave. Some caregivers can feel they have no choice in the matter, which was reported by parents (Schulz et al. 2012). Reliance on psychological or pharmacological support was crucial, highlighting the importance of screening caregivers’ wellbeing and responding to their needs (NICE 2023). This is particularly pertinent when considering how caregivers described growing to accept their situation, emphasising the role of appraisal, grounding, and recognising the daily positives, all elements that psychological therapies and peer support can assist with (Baruch et al. 2018b; Perlick et al. 1999; Proudfoot et al. 2012; Steele et al. 2010). However, it is also important to acknowledge that this acceptance may reflect a loss of hope and a lack of support from the wider mental health system. Alongside psychological support, carers require practical and financial support, alongside supportive policies to help them maintain their employment.

Social withdrawal was common among caregivers, often due to their caring commitments and a perceived lack of support or understanding from friends and family. Compared to common mental health conditions such as depression and anxiety, BD is highly stigmatised (Ellison et al. 2013; Perich et al. 2022). Many caregivers felt this stigma intensified their isolation and shame. Whilst attitudes towards BD vary globally (Latifian et al. 2023), caregivers in this review expressed a desire for greater public awareness, a need that organisations such as Bipolar UK (2022) are working to address.

Although not exclusive to BD (Cleary et al. 2020; Lillekroken et al. 2023), many caregivers felt excluded from care processes, with few noting openness from services. Key challenges included healthcare professionals providing inadequate information regarding BD and caregivers feeling unheard and uninvolved in treatment decisions, despite guidelines advocating for caregiver inclusion and information sharing (APA 2002; Malhi et al. 2015; NICE 2023; Yatham et al. 2018).

Several caregivers felt that certain structures were to blame for dissatisfaction with treatment, delayed access to support and information, and financial challenges. Whilst the UK has an Early Intervention in Psychosis pathway for first-episode psychosis (Neale and Kinnair 2017), some caregivers expressed disappointment with the lack of BD equivalent care. The Bipolar Commission has been advocating for nationwide specialist services, recognising their effectiveness in some regions (Bipolar UK 2022), however NICE (2023) guidelines do not explicitly recommend their implementation. Although these initiatives primarily target individuals with BD, it is evident that certain systemic shortcomings can result in caregivers bearing additional responsibilities (Kargar et al. 2021), a finding highlighted throughout this review. Caregivers advocated for improved collaborative practices with mental health services, including better information sharing, joint crisis planning, and access to psychological and or strategies to help manage stress. A lack of integrated frameworks for caregiver support, fragmented mental health services, limited training on caregiver inclusion, and or stretched resources contribute to caregivers’ negative experiences (Bipolar UK, 2022; Kargar et al. 2021; Ogilvie et al. 2005). Likewise, the nature of BD poses unique challenges, requiring mental health services to be both reactive and consistently responsive.

A key factor differentiating the experiences of these caregivers from those supporting individuals with other mental and or physical health conditions are the unpredictable and variable distress associated with BD (Chiao et al. 2015; El-Slamon et al. 2022; Highet et al. 2004; Kang et al. 2011; Lou et al. 2017). Caregivers were in a state of hypervigilance, monitoring any change. Some described becoming skilled in recognising early warning signs, however, this often came with feelings of being on edge and increased responsibility. Others described having limited knowledge of what to expect during and after episodes or how to respond. Transparent communication helped with this, but individuals’ perceived lack of insight into their diagnosis and non-compliance with treatment complicated matters.

Confusion around cultural differences in understanding BD symptoms may increase stress for caregivers (Rusner et al. 2012), highlighting the importance of culturally sensitive frameworks to better equip caregivers for said challenges. A Care Quality Commission (2022) review found that nearly one in six patients in UK mental health services had their ethnicity recorded as ‘not known’ or ‘not stated’, hindering services’ ability to address cultural needs. This, compounded by documented racial inequalities in BD treatment (Akinhanmi et al. 2018; Tchikrizov et al. 2023), raises concerns about whether ethnic and cultural factors receive sufficient attention in mental health care.

## Limitations

This review’s findings are based on a comprehensive and systematic search that incorporated various studies with differing methodologies and participants. Whilst offering an overview of the existing literature, we encourage readers to consult the primary studies for nuanced insights that may not be fully captured in this synthesis.

Although our findings provide valuable insights into caregivers’ experiences, the decision to only include studies conducted in Western countries with individualistic cultures limits the transferability of findings to other cultural contexts. Twenty-three studies from non-Western countries were identified and should be the focus of a future review to enable findings to be compared across different cultural contexts. Likewise, most studies did not report on participants’ ethnicities, making it difficult to determine any cultural variations. This is particularly concerning given disparities in BD diagnosis and access to mental health services among ethnic minority groups (Aggarwal et al. 2016; Bignall et al. 2019; Haeri et al. 2011).

Additionally, as this review only included studies published in English, relevant studies may have been omitted. As most participants were female, this may have influenced the findings, given documented gender differences in caregiving (Ogilvie et al. 2005; Sharma et al. 2016). Moreover, most studies did not report caregivers’ time spent supporting the individual with BD, making it unclear how findings transfer to different ‘levels’ of caregiving.

We also acknowledge imperfections in the language and framing used in the literature included in this review to reflect the experiences and challenges faced by caregivers. Caregiver burden is a recognised term within the literature and has been defined as “the level of multifaceted strain perceived by the caregiver from caring for a family member and/or loved one over time” (Liu et al. 2020). However, such terminology may suggest that the person with BD is to blame for the distress caused to the caregiver and risks distracting from the influence of interpersonal and social factors on mental distress, which is not the intention of this review. Similarly, language referring to the ‘sacrifices’ of caregivers suggests that the losses experienced are the result of choice, rather than imposed. The authors recognise that there can be complex dynamics at play in service user/carer relationships in the context of BD, and that terms like ‘caregiver burden’—which imply that the caregiver is negatively impacted by a ‘problem’ located in the service user—are not unproblematic. Furthermore, negative experiences of caregivers are often due to the lack of resources available in the wider healthcare system and beyond and there is a need for policy and practices to improve support structures for caregivers. Finally, we acknowledge that researchers’ backgrounds and beliefs may have influenced the interpretation of findings, thus we ensured to adopt a triangulation approach by having discussions with reviewers (JH & BLT) at various stages. However, the perspective of caregivers’ lived experience was missing within the research team, and thus from the analysis and interpretation of the review findings. This is a significant limitation and has implications for the wider validity of the study findings.

## Clinical and research implications

Given the challenges that caregivers face in working and communicating with healthcare professionals about diagnosis and ongoing treatment, mental health services could revisit their protocols regarding caregiver involvement. Greater involvement could reduce the responsibility and stress placed on caregivers, improve confidence to manage the unpredictable, strengthen communication with the individual, and foster trust in mental health services, ultimately bettering outcomes for all involved. As many caregivers experience high levels of stress in supporting people whose distress follows a fluctuating and sometimes unpredictable course, mental health services should proactively screen for mental distress and provide the appropriate support. As recommended in BD management guidelines (APA 2002; Malhi et al. 2015; National Collaborating Centre for Mental Health 2006; NICE 2023; Yatham et al. 2018), this could include peer support, psychological therapies, and or information on self-care strategies.

Future research could explore caregiving experiences across diverse cultural and geographical contexts to better understand any differing experiences. A broader cultural understanding would not only fill gaps in the literature but also aid in the development of culturally sensitive support tailored to the unique needs of said caregivers. Similarly, future research would benefit from examining caregiving experiences by gender as well as caregiving role (e.g., spouse, sibling), to help identify any specific needs, responsibilities, or challenges. Existing research has predominantly focused on spouses or grouped family members. Such insights could inform tailored support that better address the unique needs of different caregiver groups.

## Conclusions

This review highlights the complex experiences caregivers face when supporting individuals with BD. They report emotional, practical, financial, social, and health challenges due to the responsibilities of caregiving and the nature of the condition. Some navigated the path to acceptance and implemented strategies to manage. Caregivers are met with unhelpful and judgemental comments, with many expressing a need for greater BD awareness. Challenges collaborating and communicating with mental health services were common, with calls for improvements in service delivery. Perceived barriers with service structures and protocols decreased care satisfaction. Professional support was welcomed, and services should continue to improve screening for caregiver’s wellbeing, providing necessary support and or signposting. Services must also address caregiver-specific needs, adapting practices accordingly.

## Data Availability

The datasets analysed during the current study are available from the corresponding author on reasonable request.
